# Diet and Multiple Sclerosis: Scoping Review of Web-Based Recommendations

**DOI:** 10.2196/10050

**Published:** 2019-01-09

**Authors:** Jeffrey M Beckett, Marie-Louise Bird, Jane K Pittaway, Kiran DK Ahuja

**Affiliations:** 1 School of Health Sciences University of Tasmania Launceston Australia; 2 GF Strong Rehabilitation Research Program Department of Physical Therapy University of British Columbia Vancouver, BC Canada

**Keywords:** multiple sclerosis, diet, evidence-based medicine, internet

## Abstract

**Background:**

There is currently no scientific evidence supporting the use of specific diets in the management of multiple sclerosis (MS); the strongest dietary associations are observed with vitamin D and omega-3 fatty acid supplementation. Despite this, there are many websites that provide advice or suggestions about using various dietary approaches to control symptoms or disease progression.

**Objective:**

The objective of this study was to assess the dietary advice for the symptomatic management of MS available on the internet.

**Methods:**

This study was a systematic review of webpages that provided dietary advice for the management of MS. Webpages were selected from an internet search conducted in November 2016 using Google, Yahoo, and Bing search engines and the search term “MS diet.” The first two pages of results from each search engine were included for the initial assessment. Duplicates were removed. Data extracted from websites included specific advice relating to diet and its rationale and the citation of supporting scientific literature. Authorship and credential information were reviewed to assess webpage quality.

**Results:**

We included 32 webpages in the final assessment. The webpages made a wide variety of specific recommendations regarding dietary patterns and individual foods to help manage MS. The most common dietary pattern advised on these webpages was the low-fat, high-fiber balanced diet, followed by the low-saturated fat diet, near-vegetarian Swank diet, and the Paleo diet. The main categories of individual foods or nutrients suggested for addition to the diet were: supplements (especially omega-3 and vitamin D), fruits, vegetables, and lean protein. In contrast, the most commonly recommended for removal were saturated fats, dairy, gluten-containing grains, and refined sugar. These recommendations were often accompanied by rationale relating to how the particular food or nutrient may affect the development, prevalence and symptoms of MS; however, very little of this information is supported by the current scientific evidence between diet and MS. Only 9 webpages provided full authorship including credential information.

**Conclusions:**

There is a wide variety of Web-based dietary advice, which in some cases is contradictory. In most cases, this advice is the result of peoples’ individual experiences and has not been scientifically tested. How people living with MS use this information is not known. These findings highlight the important role health professionals can play in assisting people living with MS in their health information-seeking behaviors.

## Introduction

People are increasingly turning to the internet for health information. While health professionals remain important and trusted sources of health advice, most people search the internet as their first source of information [1-3]. The Web-based recommendations may come from respected sources such as government organizations or association websites specific to medical conditions, but increasingly, personal websites, blogs, and other forms of social media offer advice and give descriptions of individual’s experiences [4]. This easy access to information is beneficial in some respects; patients can better inform themselves about their health, and health messages can be delivered to those reluctant to engage face-to-face with health care professionals. The ability to make good health decisions based on such information forms an important part of an individual’s health literacy. This skill can be compromised if people are not able to appraise the quality, accuracy, or applicability of Web-based information. The type of information that is readily available to people in the community searching for information about diet and MS is not known.

Most people begin a Web-based search by using a search engine such as Google, Bing, or Yahoo [2]. Search engines use algorithms including, among other factors, the number of incoming links from other pages, meaning popular websites rank highly. Paid advertisements are also prominent in search results, and therefore, there is no guarantee to the scientific reliability of information found in a search [5].

Multiple sclerosis (MS) is an incurable autoimmune inflammatory disease leading to demyelination in the central nervous system, affecting approximately 2.5 million people worldwide. Presentation varies widely, with symptoms relating to neurological degeneration such as motor impairment, fatigue, pain, and sensory and cognitive disorders [6]. Around 85% of people will begin with relapsing-remitting MS, where relapses or exacerbations are followed by periods of partial or total recovery. Many of these people will eventually develop secondary progressive MS, in which there is gradual progression of the disease and incomplete recovery from relapses or exacerbations. In contrast, around 10% of people will follow a progressive course from onset, in which they suffer progressive disability in the absence of relapses or exacerbations [6]. As MS is currently incurable, treatment aims to manage symptoms, limit the frequency or severity of relapses, and slow the overall rate of disease progression. Treatments are often expensive, have varying efficacy, or involve significant side effects. As a result, many MS patients seek alternative therapies [7], including dietary modification, and the internet is the first source of information on such approaches [8,9].

There is currently no scientific evidence supporting the use of specific diets in the management of MS [10-13]; the strongest dietary associations are observed with vitamin D and omega-3 fatty acid supplementation [12]. Despite this, there are many websites that provide advice or suggestions about using various dietary approaches to control symptoms or disease progression [10], or even, as one website has claimed, “Defeating progressive multiple sclerosis without drugs” [14]. It is possible that individuals may identify alternative approaches that do offer some benefit. However, there is a risk of adopting treatments that are ineffective or even detrimental to patient health, may be arduous to follow, and also potentially expensive (spending on complementary and alternative medicines in the United States in 2007 was estimated at US $34 billion) [15].

There are many webpages providing advice about the use of diet to manage MS, despite the lack of supporting scientific evidence. This problem is exacerbated by the fact that MS patients often do not discuss information related to alternative therapies with their clinicians [8]. The aim of this study was, therefore, to determine what information people encounter from an initial internet search for MS-related dietary advice, its rationale, and sources.

## Methods

This review of Web-based dietary advice for the management of MS symptoms was performed in accordance with a protocol described previously [16], with some minor amendments. Briefly, Google, Yahoo, and Bing search engines were used within a new Incognito window on the Chrome browser to conduct a search using the term “MS diet.” The search was performed by a single investigator (MLB) in November 2016 and included no search limitations (eg, date). The first 2 pages of results from each search engine were included for initial assessment. Each webpage that resulted from this search strategy was archived using the FireShot extension for Chrome, for later assessment. The initial webpage linked-in search results, and any other relevant webpages within the same website, were included as one result. Duplicate results between search engines were removed, and any advertisements with no relevance to MS were excluded. Scientific papers on journal publisher websites were excluded as they are often not free to access and people without scientific training may lack the knowledge to properly interpret the findings of the paper. It would also be inappropriate to depend on results of individual studies. Other relevant links (related to the topic of MS diet) from webpages found in the primary search were included for assessment if they met the inclusion criteria.

Data from the included webpages were extracted by two reviewers (KDKA and JKP) in January 2017. Each included source was classified by the type of webpage and the type of website that it was published on. A webpage was defined as an individual document displayed by a browser; a collection of webpages grouped together was defined as a website. Webpages were classified as articles or blogs. Articles were documents presenting information but with no provision for publishing comments, feedback, or discussion from readers. Blogs were considered as webpages that may have presented information in a similar manner to articles but allowed for readers to add comments or questions on the same webpage. Initially, the information on the “About” page of each website was intended to be used to help determine the website classification; however, not all websites provided useful information. Websites were therefore classified by assessing the source of the information (government-endorsed vs nongovernment-endorsed website) and whether it was an MS-specific or a general health information site. Websites were classified as government websites, nongovernment general health websites, nongovernment MS-only websites, and personal websites.

Data were extracted for the date of last update, country of origin, provision of specific advice relating to diet and its rationale, and the citation of supporting scientific literature. Webpages were assessed to determine if they provided specific guidance to consult a health professional in relation to the dietary advice given; general website legal disclaimers were not included. During data extraction, webpage authorship was assessed as NA (author information not available on the webpage), A (only author’s name provided on the webpage), or FA (full authorship: author’s name and relevant credentials provided).

## Results

**Figure 1:**
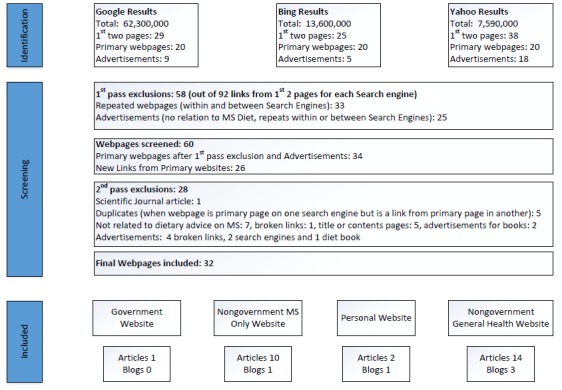
Preferred Reporting Items for Systematic Reviews and Meta-Analyses flowchart for the selection of webpages. MS: multiple sclerosis.

Searches using the term “MS diet” returned 62,300,000 results for Google; 13,600,000 for Bing; and 7,590,000 results for Yahoo. The first 2 pages from these sites contained links to 25-38 pages (Figure 1), making a total of 92 webpages. At the first pass check for duplicates within, and between, search engines and for advertisements not related to MS diet (eg, Weight Watchers), 58 webpages were removed. Screening of the remaining 34 pages generated an additional 26 new webpages (criteria—listed under sources). During the second pass, we removed 28 webpages (including advertisements, broken links, duplicate pages, scientific journal articles, and pages not relevant to MS diet), leaving 32 webpages for data extraction.

The year of the last update was included in 25/32 webpages. The oldest recorded update was dated 2011, and the most recent was dated 2016. Most of these webpages (n=19) listed the United States in their contact details, followed by Australia (n=6) and the United Kingdom (n=4). We were unable to identify the country of origin for 4 webpages. Targeted advertisements were included in 12 webpages.

The 32 webpages assessed (Table 1) came from four different types of websites including government websites (1 article); nongovernment MS-only websites (10 articles and 1 blog), personal websites (2 articles and 1 blog), and nongovernment general health websites (14 articles and 3 blogs). The single government website [17] included a scientific overview of MS discussing the types of MS, symptoms, treatment, and alternative therapies including diet that may or may not be effective. This webpage did not include any information on authorship and did not provide any direct link to scientific citations.

Nongovernment MS-only websites included 10 webpages from advocacy, not-for-profit organizations including MS Australia (4 webpages), MS Society UK (1 webpage), National MS Society USA (2 webpages), Swank MS foundation (1 webpage), Overcoming MS (1 webpage), and Direct MS (1 page). There was 1 webpage (MS Discovery Forum) that was designed as a Web-based resource and discussion forum for researchers, rather than for the general public. Only 1 webpage [18] provided full authorship information.

**Table 1:** Webpages included in data extraction.

Type		Title	Authorship
**Government websites**			
	Article	Multiple Sclerosis (MS) [17]	NA^a^
**Nongovernment MS-only websites**			
	Article	Australian researchers find ‘bad’ fats major culprit in MS progression [19]	NA
	Article	High salt diet linked to autoimmunity^b^ [20]	NA
	Article	New dietary research looks into fatty acids, vitamins, and lipids in MS^b^ [21]	NA
	Article	Fish and flaxseed oil linked to improved quality of life and MS disease activity^b^ [22]	NA
	Article	Treatment of MS via Diet^b^ [23]	NA
	Article	Diet & Nutrition [24]	NA
	Article	Diet-live it well [25]	NA
	Article	Overcoming Multiple Sclerosis-diet [18]	FA^c^
	Blog	Multiple Sclerosis Discovery Forum: Does diet matter in MS? [26]	A^d^
	Article	Diet [27]	NA
	Article	Roger MacDougall’s Story [28]	A
**Personal websites**			
	Article	Multiple Sclerosis and food hypersensitivities [29]	A
	Blog	The MS Diet-MS Diet for Women [30]	A
	Article	Suggestions for the Newly Diagnosed [31]	A
**Nongovernment general health websites**			
	Article	Treating Multiple Sclerosis with Diet: Fact or Fraud? [32]	FA
	Article	MS foods to avoid [33]	FA
	Article	Everyday health: 7 foods to avoid when you have MS [34]	FA
	Article	Medical Daily: Multiple Sclerosis diet: Doctor Terry Wahl’s reverses MS with diet alone [35]	A
	Article	Is there a Multiple Sclerosis diet? [36]	NA
	Article	An MS-Stricken Doctor Changes Her Diet [37]	A
	Article	Dr. McDougall’s Health & Medical Centre: Diagnosed with MS, food became my medicine [38]	A
	Article	What to eat when you have Multiple Sclerosis [39]	A
	Blog	How to treat Multiple Sclerosis with diet [40]	FA
	Article	Dietary therapies for MS [41]	FA
	Blog	The MS recovery diet [42]	NA
	Article	Multiple Sclerosis & dietary intervention [43]	A
	Article	The Best Bet Diet for MS [44]	FA
	Article	My experience with Multiple Sclerosis and my route to becoming symptom-free [45]	A
	Article	Swank Diet information [46]	A
	Article	Does Your Diet Affect Your MS? [47]	A
	Blog	Doctor reverses MS in 9 months by eating these foods [48]	FA

^a^NA: author information not available on the webpage.

^b^Webpage no longer available (checked on July 14, 2018).

^c^FA: full authorship, author’s name, and relevant credentials reported on the webpage.

^d^A: author’s name given on the webpage.

There were 2 personal websites by people living with MS about the diets they were following and that they claimed to work for them. The third personal website presented a reproduction of an article written in the style of a scientific research article, which included the pathogenesis and potential environmental and dietary factors associated with MS, authored by a relative of a person living with MS. None of the authors fitted the criteria for full authorship. Nongovernment general health websites included Web-based health magazines with webpages on MS and diet and websites on specific diets such as the Paleo diet and its purported association with MS. Of the 17 websites, 7 webpages provided full authorship information.

Social media links enabling the sharing of information from the individual webpages were included on all sites. While most websites contained disclaimers or legal terms and conditions indicating that the information provided did not constitute medical advice, only 11 webpages included specific advice to consult a medical practitioner, neurologist, health care provider, or dietitian before making any changes to the diet.

The webpages made specific recommendations regarding dietary patterns (Table 2) and individual foods to help manage MS (Tables 3, 4, 5, and 6). The most common dietary pattern (Table 2) advised on these webpages was the healthy balanced diet (low fat and high fiber) based on American Heart Association recommendations (7 webpages), followed by the low-saturated fat, near-vegetarian Swank diet, including the Overcoming MS and MS Recovery diets (6 webpages); the Paleo diet (low processed, low-grain foods) and its modifications, including Wahl’s diet (4 webpages); and Ashton Embry’s Best Bet diet and its modifications (2 webpages). Combining the information from dietary patterns and individual food recommendations, the main categories of individual foods or nutrients suggested for addition to the diet were: supplements (especially omega-3 and vitamin D), fruits, vegetables, and lean protein (including skinless poultry, grass-fed meat, and organ meat; Tables 4 and 5). In contrast, the most commonly recommended for removal were fats (saturated, heated, etc), dairy, gluten-containing grains, and refined sugar (Tables 3 and 6). These recommendations were often accompanied by rationale relating to how the particular food or nutrient may affect the development, prevalence, and symptoms of MS (Tables 2, 5 and 6).

**Table 2:** Recommendations and rationale for the dietary patterns recommended by the websites.

Dietary pattern	Major characteristics	Rationale for this diet
Healthy balanced diet	Low-fat, high-fiber diet with whole grains and fish (similar to the diet recommended by the American Heart Association)	Increases the time between relapses and promotes overall health [24,25,34]; controls weight and fatigue, better bladder and bowel function [24,25]; reduces inflammation [47]; low vitamin & mineral intake can worsen multiple sclerosis (MS) symptoms [36]
Swank diet and its modifications including Overcoming MS and MS Recovery diet	Low-saturated fat, near-vegetarian diet with no red meat in the first year; dairy with <1% fat; no processed foods; saturated fat <15 g/day; unsaturated fat 20-50 g/day; cod liver oil and multivitamin every day	Low prevalence in population on low-saturated fat diets [32], low-saturated fat, near-vegetarian diet arrests or cures MS and slows progression [38]; fruits and vegetables reduce constipation and reduce weight [32]; lower frequency and severity of attacks [40]; better health outcomes; cow milk protein is similar to myelin and initiates autoimmune reaction in MS [18]; Swank diet reduces death rate [46]
Paleo diet and its modifications including Wahl’s diet	Includes free-range meat and organic fruit and vegetables. Excludes grains, dairy, soy, legumes, and sugar	For optimum mitochondrial, myelin, and neurotransmitter functions [35,48]; to slow MS decline [35]; animal-based omega-3 to lessen progression and relapses [35]; seaweed for iodine, iron, calcium, and fiber helps increase alertness and mental clarity [35,48]; vitamins A, B, C, and K for myelin and brain health [27,48]; sulfur-rich vegetables for removing toxins and formation of neurotransmitters [27,48]; colorful fruits and vegetables for antioxidants [37]; grains are health destroying [37]; omega-3, creatine, and coenzyme Q10 help in mitochondrial function [48]
Best Bet diet	Includes vitamin, mineral, and herb supplements. Excludes dairy, refined sugar, eggs, yeast, gluten, and legumes	Remove proteins that resemble myelin [44] and act like allergens [45]

**Table 3:** Number (proportion) of recommendations to remove foods.

Foods to remove	Recommendations^a^ to remove foods (n=79), n (%)
Fats	16 (20)
Dairy	12 (15)
Sugar	10 (13)
Grains (gluten)	10 (13)
Eggs	7 (9)
Caffeine and Alcohol	5 (6)
Condiments (including salt)	4 (5)
Meat and Poultry	4 (5)
Legumes	4 (5)
Processed Food	3 (4)
Yeast	3 (4)
Fruit (citrus)	1 (1)

^a^Some webpages made multiple recommendations for addition or removal of foods from the diet; therefore, number of recommendations may be greater than the number (32) of included webpages.

**Table 4:** Number (proportion) of recommendations to add foods.

Foods to add	Total recommendations^a^ to remove foods (n=62), n (%)
Fruits	11 (18)
Vegetables	11 (18)
Supplements	10 (16)
Fish	6 (10)
Meat and Poultry	5 (8)
Other Grains	4 (6)
Oils and unsaturated fats	3 (5)
Condiments (including salt)	3 (5)
Nuts and Seeds	2 (3)
Dairy Alternatives	2 (3)
Lentils and Legumes	2 (3)
Water	2 (3)
Eggs	1 (2)

^a^Some webpages made multiple recommendations for addition or removal of foods from the diet; therefore, number of recommendations may be greater than the number (32) of included webpages

**Table 5:** Recommendations and rationale provided by the websites for foods or nutrients to be included in the diet.

Foods or nutrients to add to diet	Foods to add	Rationale for change
Drinks	Plenty of water	Neuron activity and brain functionality [30]
Supplements	Fish oil, B1, B2, B12, biotin, iodine, vitamin A, vitamin E, creatine, coenzyme Q10, antioxidants, probiotics, vitamin D, omega-3, omega-6, evening primrose oil, magnesium, vitamins, and minerals	Omega-3, vitamin D: reduce frequency of attacks [18,21,22,46]; omega-3 an essential fatty acid as these fatty acids make myelin [31]; vitamin D: regulator of immune system [31]; vitamin B12: helps reduce exacerbation of multiple sclerosis (MS) [31]; low vitamin D: development of MS [39,48]; low vitamin D: more aggressive progression [39]; evening primrose and fish oil supplements to reduce severity and length of attacks [17]; omega-3 and -6 and vitamin A and E combined delay time to progression [23]
Egg	White only	N/A^a^
Meat and poultry	Grass-fed, organ meat, lean, skinless chicken	As a replacement for high-fat meats [28]; lean protein to combat fatigue [29]
Lentils and legumes	N/A	N/A
Oil or unsaturated fats	Olive, sunflower, safflower, flaxseed, fish	As a replacement for animal fats [28]; extra virgin olive oil rich in omega-9; anti-inflammatory [30]; fish oil reduces progression [30]
Other grains	Whole grains, brown rice	N/A
Fish	Oily fish, seafood	Build up and repair myelin sheath, reduce inflammation, decrease certain immune reactions, improve relapse, slow progression, and improve MS symptoms [30]; lower relapse rate [22]; slows progression and less relapse [35]
Nuts and seeds	Flaxseed	Energy and nutrients for stable blood sugar and omega-3 rich for improved metabolism [30]; lower relapse rate [22]
Condiments	Himalayan pink salt, sea salt, fermented vegetables, seaweed	Fermented vegetables for gut bacteria and health [48]; seaweed for iodine, iron, calcium, and fiber help increase alertness and mental clarity [35,48]
Dairy alternatives	Rice milk, almond milk, soy milk	Replacement for dairy [30]
Fruit	Berries, brightly colored, antioxidant rich, raw	High in antioxidants, but no link provided to symptoms [30]; high in vitamins, minerals, and antioxidants for optimal mitochondrial, myelin, and neurotransmitter functions [35,48]
Vegetables	Green, brightly colored, white, raw	Immune system health [30]; reduce inflammation [30]; high in antioxidants to help fight MS symptoms [30]; high in vitamins, minerals, and antioxidants for optimal mitochondrial, myelin, and neurotransmitter functions [26,48]; vegetables rich in antioxidants and vitamins help reducing toxins and creating neurotransmitters [37]

^a^N/A: not applicable.

**Table 6:** Recommendations and rationale provided by the websites for foods or nutrients to be removed from the diet.

Foods or nutrients to remove from the diet	Foods to remove	Rationale for change
Fats	Saturated fat, heated fats, margarine, trans fats, fatty foods, coconut oil, palm oil, animal fats, cholesterol, highly marbled meat	High blood cholesterol and LDL^a^: greater number of new lesions [19,21], higher rate of disability progression [19]; saturated fat: inflammation [18] and breaches blood-brain barrier, which precedes immune hypersensitivity in the CNS^b^ [30]; heated fats (fried food): cannot be absorbed and cause damage to cells [30]; margarine contains trans fats and causes inflammation [30,34]; fried food hard to digest [28]; trans fats and cyclic fats embed in cell membrane and distort cellular function [48]; high fats may be a risk factor for multiple sclerosis (MS) development [26]; development of MS [18,39]; slower progression of MS [18,32]; prevalence [29]; MS patients at high risk of CVD^c^: saturated fat increases risk [34]
Dairy	Cow’s milk, full-fat milk, pasteurized milk, butter fat, casein	Development of MS [30]; prevalence [29,30,48]; to improve overall health [33]; allergy leading to attack [28,45]; may be detrimental [34]; aggravates condition [42]; immune hypersensitivity and cross reactivity [18,29,44]
Grains	Gluten-containing foods, processed grains, starches, cereals	Causing autoimmune response [29,30]; grains are totally health destroying [37]; prevalence [29]; MS population at high risk of celiac disease [34]; processed carbohydrates leading to high blood sugar and CVD [34]
Sugar	Refined, artificial sweeteners, fructose, sweetened and fizzy drinks	Fatigue [33,34,39]; weight [34]; inflammation and regulation of immune system by insulin (due to sugar) and artificial sweeteners, candida overgrowth leading to leaky gut syndrome [30]; development of MS [48]; increase uric acid, which increases inflammation [48]; aspartame metabolizes to methanol, which is a potent neurotoxin [48]; artificial sweeteners can irritate bladder [39]; sugary snacks can cause energy crash [39]
Legumes	N/A^d^	Can cause reaction and lectin from green beans can reduce absorption of certain nutrients [30]; immune hypersensitivity [29]
Meat and poultry	Land animals, highly marbled meat	To reduce saturated fat [30]
Eggs	Egg yolks	To reduce saturated fat [30]; immune hypersensitivity [29]; allergic reaction [45]
Fruit	Citrus	May affect MS symptoms [30]
Condiments	Salt	Dietary sodium may be a risk factor and may also exacerbate disease activity [20,26]; worsen symptoms [20]; high sodium associated with poor prognosis [39]; risk of relapse and new lesions [34]; risk factor for heart disease [34]
Drinks	Alcohol, whiskey, gin, vodka, caffeine	Caffeine: blocks adenosine receptors, hence lowering the effect of adenosine in suppressing inflammation [30]; to avoid insomnia [39]; alcohol intensifies feelings of fatigue [36]
Yeast	N/A	Immune hypersensitivity [29]
Processed foods	N/A	None provided

^a^LDL: low-density lipoprotein.

^b^CNS: central nervous system.

^c^CVD: cardiovascular disease.

^d^N/A: not applicable.

## Discussion

Predictably, there is a wide range of dietary advice solicited on the internet for treating MS and its symptoms. The unexpected result was that the advice not only ranged from recommendations for the addition or removal of individual foods and nutrients but also to broader changes affecting entire dietary patterns, with two dominant and significantly different patterns emerging. These were a Paleo-style diet, low in processed and grain foods but including animal fats such as lard, and the low-fat, near-vegetarian Swank diet. The advice was generally poorly backed by scientific evidence and often purported by people who claim to have controlled or even reversed their MS symptoms by making changes to their diet.

There is only weak scientific evidence for a relationship between diet and MS [10,12]. Associations such as the MS Societies, and government health sites, perhaps considered as the most esteemed places to look for advice, have taken this on board and appropriately recommend a low-fat, high-fiber, healthy balanced diet. While this approach is in line with the scientific evidence, the information on these websites is often presented in a bland and matter-of-fact manner with little or no authorship, author credential information, or citation of supporting scientific evidence. This is despite the fact that the public is often advised that webpage credibility should be judged on transparency and the inclusion of authorship, credentials of the authors, and citation of scientific literature [49,50]. Admittedly, these websites are commonly written by a team rather than an individual and include little or no scientific jargon or citations in an attempt to make the information easier to understand. This, however, may lead to unintended consequences for the message and its credibility.

In contrast to the advocacy sites, personal websites are often visually attractive and as well as including authorship, the author possesses the credential of “MS sufferer,” making it a site for and about people who know the disease intimately. These sites often include citations to the scientific literature, which may or may not have supported their claims, but their presence can potentially still give credibility to the site or person. Nongovernment general health websites (more like internet magazines) commonly include authorship and the credentials of the writers (often a person with medical or nutrition background) and opinions from people living with MS who claimed to have “tried and tested” a number of approaches. These people report on what they felt worked best for them to control their MS symptoms and, more importantly, encourage others to make similar changes and assess the suitability for themselves. This encouragement and the suggestion to individuals to tailor their diet to suit themselves may potentially lead to a feeling of improved self-worth, by having a greater input in their own treatment.

Webpages that advise consuming a healthy balanced diet do so without reference to scientific literature and simply state that there is no evidence for adopting a specialized diet for MS. In contrast, webpages that gave recommendations to change individual foods or nutrients, or entire dietary patterns, attempted to provide a rationale for the changes and often some citation of scientific literature. This approach, regardless of how strongly the advice might be supported by scientific evidence, potentially provides a more compelling case to the reader to try these alternative diets. On webpages that suggested the addition or removal of specific foods or nutrients in the diet, there was commonly an explanation provided as to what benefit the change would provide in relation to MS symptoms or the underlying mechanisms. Often, recommendations to remove a food were given alongside alternative foods that could be used as substitutes. For example, dairy products were commonly recommended for elimination from diets because it was reported that milk proteins mimic parts of the myelin sheath protein, leading to autoimmune reactions [28], and suitable alternatives such as rice and almond milk were suggested [30]. Recommendations to change dietary patterns took a similar approach by attempting to explain the rationale behind the dietary changes. The Wahl’s Paleo diet recommended consumption of 3 cups of leafy green vegetables daily because it was considered that they are rich in vitamins A, C, K, and B and various minerals, which are essential for brain function and the protection of mitochondria.

Given the complexities of many diseases, individually tailored approaches to treatment may be more effective, and a number of writers on webpages included in this study did advise trialing their suggested changes to see whether they were effective for the individual. If the level of success in treating MS symptoms using dietary approaches is similar to that suggested by these internet sources, the scientific community perhaps needs to work toward testing the basis of some of the dietary approaches that have become popular in this population, such as the Swank and Wahl’s diets.

The desire for many patients to follow an alternative approach to try to improve their health cannot be ignored by clinicians. The internet is a readily accessible source of advice and information for patients; however, the advice could be ineffective or even detrimental to the patient’s health. It is known that patients frequently do not actively seek to discuss alternative therapies with their clinician [8], and as evidenced in this study, the information available about such therapies can vary considerably. Clinicians should seek to open a dialogue with their patients to determine if they are considering alternative therapies and help to direct them to reliable sources of information. By doing this, health professionals can improve a person’s health literacy and assist them in appraising information and empower them to make appropriate health decisions [51].

The search strategy was designed to be similar to an initial search that a member of the public, with no particular training, may undertake. Therefore, we sought to use a simple search term and popular search engines. It could potentially be considered that using a single search term such as “MS diet” was a weakness of the study design. However, the suitability of this term was informed by a pilot study using Google Trends [52], where “MS diet” was the top trending search query on this topic between March 2010 and March 2016. Other proposed search terms were found to have very low numbers of search queries on Google Trends, indicating that they were not commonly used search terms and, therefore, were not included in the study. Similarly, the decision to limit the search to the first two pages of search results was based on reported behavior analysis indicating that the first page of search results receives approximately 92% of all traffic and the second page perhaps as little as 5% of traffic [53]. Including results beyond these two pages would, therefore, mean including pages that were rarely visited in most everyday searches. We acknowledge that social and patient networks are likely to become important sources of information as the patient or carers search more intensely and change their information-seeking approach. The inclusion of only English language websites may also be considered as a limitation.

The search strategy used an Incognito browser to avoid the influence of past searches on our results, but geolocationing was not disabled. This may affect the generalizability of our results; however, we note that only 16% (5/32) of included webpages had an Australian domain. Information on the internet is continually evolving, and the total number of possible search hits is often very large. However, we do not consider that this affects the validity of the data presented as the aim of the study was to review the webpages that were most likely to be accessed on an initial search.

In conclusion, there is a wide variety of Web-based dietary advice that, in some cases, offers contradictory advice. In many cases, this advice is the result of peoples’ individual experiences and has not been scientifically tested. The public is advised to assess the reliability of health information provided on the internet by looking for details such as authorship, and supporting evidence, such as the citation of scientific literature. However, we found webpages that would normally be considered reliable (eg, MS Society) did not perform well in this regard. Conversely, some webpages appeared credible due to the provision of links to scientific literature, but the cited material did not always support the advice given. Patients without scientific training, and likely to lack the knowledge required to interpret the conflicting and often unsupported advice given, are left to assess whether the information being provided is reliable. These findings highlight the role health professionals can have in providing the best quality information to consumers on relevant topics in a way that is easy to understand, accurate, and accessible. Future work should focus on determining what decisions people make from accessing Web-based information and the proportion of people who actually follow the different dietary regimes promoted on the Web, what factors led them to choose that particular approach, and what, if any, effects (positive or negative) have resulted from making these dietary changes.

## Abbreviations

MS: multiple sclerosis
